# MicroRNA-137 inhibits the inflammatory response and extracellular matrix degradation in lipopolysaccharide-stimulated human nucleus pulposus cells by targeting activin a receptor type I

**DOI:** 10.1080/21655979.2022.2042987

**Published:** 2022-03-02

**Authors:** Bin Yu, Ziqi Zhu, Beiduo Shen, Jiawei Lu, Kai Guo, Weidong Zhao, Desheng Wu

**Affiliations:** Department of Spine Surgery, Shanghai East Hospital, School of Medicine, Tongji University, Shanghai, China

**Keywords:** microRNA-137, ACVR1, intervertebral disc degeneration, lipopolysaccharide

## Abstract

This study aimed to investigate the role played by microRNA (miR)-137 in intervertebral disc degeneration via targeting activin A receptor type I (ACVR1) and the underlying mechanism. Human nucleus pulposus cells were exposed to 10 ng/mL lipopolysaccharide (LPS) to establish an *in vitro* intervertebral disc degeneration model. ACVR1, extracellular matrix degradation-associated genes (aggrecan and collagen type II) and miR-137 levels were assessed using reverse transcription-quantitative polymerase chain reaction (RT-qPCR) and Western blotting assays. The MTT (3-(4,5-dimethyl-2-thiazolyl)-2,5-diphenyl-2-H-tetrazolium bromide) assay and flow cytometry were used to evaluate nucleus pulposus cell viability and apoptosis. Additionally, the association between miR-137 and ACVR1 was predicted and verified using bioinformatic software and dual-luciferase reporter assays. Furthermore, the secretion of inflammatory factors was analyzed via enzyme linked immunosorbent assay (ELISA). Our results confirmed that ACVR1 was upregulated in lipopolysaccharide-treated nucleus pulposus cells. Lipopolysaccharide suppressed cell viability, promoted apoptosis, enhanced the secretion of inflammatory factors, and reduced aggrecan and collagen type II expression. However, these results were reversed upon ACVR1 silencing. Our data revealed that ACVR1 directly targets miR-137 and is negatively regulated by miR-137 in nucleus pulposus cells. Additionally, the miR-137 mimic promoted cell growth, reduced cell apoptosis, reduced the secretion of inflammatory cytokines, and accelerated extracellular matrix accumulation in lipopolysaccharide-exposed nucleus pulposus cells. However, ACVR1 plasmid abolished the functions of the miR-137 mimic in lipopolysaccharide-exposed nucleus pulposus cells. Together, these findings indicate that miR-137 suppresses the inflammatory response and extracellular matrix degradation in lipopolysaccharide-treated nucleus pulposus cells by targeting ACVR1.

## Graphical Abstract

**Figure:**
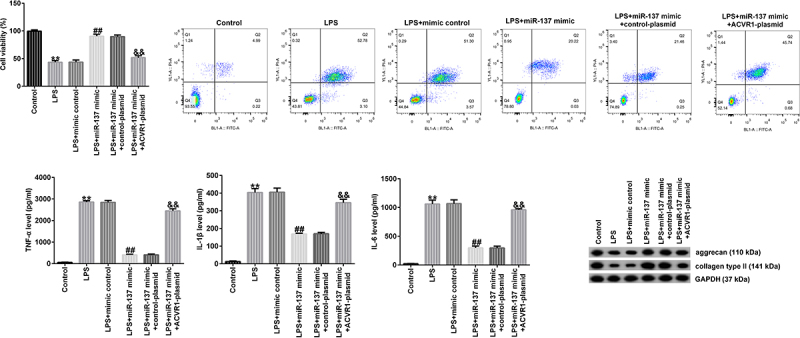

## Introduction

Intervertebral disc degeneration (IDD), a chronic, complex, and multifactorial musculoskeletal disorder, is a major cause of lower back pain that results in disability and poor quality of life [1,2]. Importantly, >80% of the elderly suffer from IDD, which has become a complicated issue due to economic loss and burdensome demand on healthcare systems [3]. Recent reports have shown that various factors, including stimulation of pro-inflammatory factors, alteration of the intervertebral disc cell phenotype, and extracellular matrix (ECM) degradation, change during IDD [4,5]. However, the nosogenesis of IDD is intricate, and effective treatments for IDD remain limited. Previous reports have suggested that protection against nucleus pulposus (NP) cell apoptosis and NP cell activation can block IDD development [6]. Moreover, in previous studies, lipopolysaccharide (LPS) was used to stimulate the production of inflammatory cytokines and ECM degeneration in NP cells *in vitro* [7,8].

Numerous studies have confirmed that activin A receptor type I (ACVR1) functions as a vital receptor for bone morphogenetic proteins (BMPs) and regulates the pathogenesis of diseases. For instance, Wang et al. illustrated that downregulation of ACVR1 accelerates osteogenesis by regulating the Wnt signaling pathway [9]. A previous study demonstrated that ACVR1 is markedly overexpressed in patients with IDD [10]. Nevertheless, the action mechanisms of ACVR1 in IDD and its downstream or upstream microRNA (miRNA or miR) regulators remain unclear. Thus, this study aimed to illustrate the functions and mechanisms of ACVR1 in IDD.

miRNAs are highly conserved RNAs (22 nucleotides) that negatively regulate mRNA translation by targeting the 3ʹ-UTR of the target mRNA [11]. miRNAs are associated with numerous biological processes, including cell proliferation, apoptosis, and senescence and inflammatory diseases [12–14]. Various reports have shown that certain miRNAs can play wide roles, including NP cell apoptosis, inflammatory reactions, and ECM metabolism, in the occurrence of IDD [15–17]. For example, Zhao et al. revealed that miRNA-143 regulates apoptosis in human IDD NP by targeting BCL2 [15]. Moreover, an investigation by Li et al. suggested that an miR-27b inhibitor reduces type II collagen levels by targeting MMP13 in IDD [16]. MiRNA-137 has been reported to be involved in various diseases such as cancer, epilepsy, and neuropathic pain [18–20]. A recent study reported that miR-137 is downregulated in LPS-induced chondrocytes and articular cartilage samples, and miR-137 reverses the development of osteoarthritis (OA) by targeting the suppression of TCF4 via the AMPK/NF-κB signaling pathway [21]. Nevertheless, the specific function and latent mechanism of miR-137 in IDD remain unclear.

It is worth mentioning that in a bioinformatic software assay, it was noted that ACVR1 potentially targets miR-137. Therefore, we hypothesized that miR-137 participates in the occurrence and progression of IDD by mediating the expression of ACVR1.In recent years, human NP cells stimulated by LPS have been recognized as an *in vitro* model for IDD research [22–24]. Therefore, in this study, we explored the role of ACVR1 and miR-137 in LPS-stimulated human NP cells to illustrate the functions and potential mechanisms of action of ACVR1/miR-137 in IDD.

## Materials and methods

### Cell culture and LPS treatment

The present study was authorized by the Ethics Committee of Shanghai East Hospital, Tongji University School of Medicine (approval number: 2019–035). NP cells were purchased from Procell Life Science & Technology Co., Ltd. (cat. CP-H097; Procell Life Science & Technology Co., Ltd., Wuhan, China; https://www.procell.com.cn/view/2246.html) and seeded in 25 cm culture flasks at a density of 1 × 10^6^. The cells were cultivated in Dulbecco’s Modified Eagle Medium (DMEM)/F12 (Gibco, Grand Island, NY, USA), supplemented with 1% streptomycin and 15% FBS (Gibco), in a cell incubator maintained at 5% CO_2_ and 37°C. In this experiment, NP cells were treated with 10 ng/mL LPS (Sigma-Aldrich; St. Louis, MO, USA) for 24 h to set up an *in vitro* IDD cell model [25].

### Dual luciferase reporter assays

Bioinformatic analysis was conducted using TargetScan version 7.2 to predict miR-137 target genes. ACVR1 harbors putative miRNA-137 binding sites. Wild-type (WT: 5ʹGGUGAAUUUUUAAUCAGCAAUAU3’) or mutant type (MUT: 5ʹGGUGAAUUUUUAAUCUCGUUAUU3’) 3’-UTR sequences of ACVR1, which includes the miRNA-137 binding site or a mutated target site, was amplified and cloned into the pGL-control vector (Promega Corporation, WI, USA) to obtain the ACVR1-WT or ACVR1-MUT constructs using PmeI, XbaI and NotI restriction enzymes. To point-mutate the miR-137 binding domain on the 3ʹUTR of ACVR1, a QuikChange Site-Directed Mutagenesis Kit (Stratagene; Agilent Technologies, Inc.) was performed following the manufacturer’s instructions. The luciferase reporter vectors were verified by sequencing. Next, NP cells were co-transfected with the control-mimic or miR-137 mimic (Shanghai GenePharma Co., Ltd.) along with luciferase reporter vectors using Lipofectamine 2000 (Invitrogen; Thermo Fisher Scientific, Inc.) following the manufacturer’s protocol. After 48 h, the cells were collected, and luciferase activity was assessed using a Dual Luciferase Reporter Assay System (Promega Corporation) according to the manufacturer’s protocol [26].

### Cell transfection

Mimic-control (5-ʹUUCUCCGAACGUGUCACGUTT-3ʹ) and miR-137 mimic (5ʹ-UUAUUGCUUAAGAAUACGCGUAG-3ʹ) were obtained from Guangzhou RiboBio Co., Ltd. (Guangzhou, China). The control-siRNA (cat no. sc-36869), ACVR1-siRNA (cat no. sc-40202), CRISPR activation control plasmid (control plasmid; cat no. sc-437275), and ACTR-ICRISPR Activation Plasmid (ACVR1-plasmid; cat no. sc-401283-ACT) were obtained from Santa Cruz Biotechnology, Inc. (Dallas, LX, USA). Human NP cells were transfected with 100 nM mimic-control, 100 nM miR-137 mimic, 0.2 μM control-siRNA, 0.2 μM ACVR1-siRNA, 1 μg control-plasmid, 1 μg ACVR1-plasmid, 100 nM miR-137 mimic+1 μg control-plasmid, or 100 nM miR-137 mimic+1 μg ACVR1-plasmid using Lipofectamine 2000 (Invitrogen) according to the manufacturer’s instructions. After 24 h of transfection, the cells were used for subsequent experiments.

### Enzyme-linked Immunosorbent Assay (ELISA)

The secretion of tumor necrosis factor (TNF)-α (human TNF-α ELISA kit; cat no. PT518), interleukin (IL)-1β (human IL-1β ELISA kit; cat no. PI305), and IL-6 (Human IL-6 ELISA kit; cat no. PI330) in the NP cell culture supernatant was quantitatively assessed using ELISA kits (RayBiotech, GA, USA) according to the manufacturer’s instructions. The OD values at 450 nm were assessed using a Multiskan Spectrum (Molecular Devices, LLC, Shanghai, China) [27].

### MTT assay

NP cells were incubated at 37°C for 24 h, transfected with control-siRNA, ACVR1-siRNA, mimic-control, miR-137 mimic, control-plasmid, or ACVR1-plasmid for 24 h, and exposed to 10 ng/mL LPS for another 24 h. Subsequently, the cells were exposed to 10 μL MTT solution and cultured at 37°C for 4 h. The formazan product was dissolved in dimethyl sulfoxide. Finally, a microplate reader (BioTek, USA) was used to detect the OD values at 570 nm [28].

### Flow cytometric analysis

After transfection and LPS treatment, the cells were stained using an annexin V-fluorescein isothiocyanate (FITC)/propidium iodide (PI) apoptosis detection kit (BD Biosciences) following the manufacturer’s instructions. Briefly, NP cells were stained with 5 μL Annexin V-FITC and 5 μL PI at 4°C for 30 min in the dark. Next, flow cytometry (BD Biosciences) was performed to detect apoptotic cells, and the data were analyzed using CellQuest software version5.1 (BD Biosciences) [29].

### Reverse transcription-quantitative PCR (RT-qPCR) analysis

Total RNA was extracted from NP cells, using an RNA isolation kit (Invitrogen, Carlsbad, CA, USA) following the manufacturer’s instructions. DNase treatment was conducted to digest contaminating genomic DNA and RNA quality was detected by A260/A280 absorbance using a NanoDrop. RNA (2 μg) was then reverse transcribed into cDNA using a Reverse Transcription Kit (Fermentas; Thermo Fisher Scientific, Inc.) according to the manufacturer’s instruction. The reverse transcription reaction conditions were as following: 25°C for 5 min, 42°C for 60 min and 80°C for 2 min. SYBR Premix Ex Taq (Takara, China) was used to determine gene expression levels following the manufacturer’s protocol. The following thermocycling conditions were used for qPCR: Initial denaturation at 95 °C for 5 min; followed by 38 cycles of 15 sec at 95 °C, 1 min at 60 °C and 30 sec at 72 °C; and a final extension for 10 min at 72 °C. The relative expression of target genes was calculated using the 2^−ΔΔCq^ method [30]. Experiments were repeated for three times. The primer sequences were as follows:

U6 forward, 5’-GCTTCGGCAGCACATATACTAAAAT-3 ’

reverse, 5’-CGCTTCACGAATTTGCGTGTCAT-3’

GAPDH forward, 5’-CTTTGGTATCGTGGAAGGACTC-3v

reverse, 5’-GTAGAGGCAGGGATGATGTTCT-3’

miR-137 forward 5’-ACACTCCAGCTGGGTTATTGCTTA AGAATAC-3’

reverse 5’-TGGTGTCGTGGAGTCG-3’

ACVR1 forward 5’-TTCGCCGGAGAAGGACTC-3’

reverse 5’-GCTGCATAGCAGATTTGGGC-3’

aggrecan forward, 5’-CTACCAGTGGATCGGCCTGAA-3’

reverse, 5’-CGTGCCAGATCATCACCACA-3’

collagen type II forward, 5’-GGCAATAGCAGGTTCACGTACA-3’

reverse, 5ʹ-CGATAACAGTCTTGCCCCACTT-3ʹ

### Western blot analysis

Total proteins from NP cells were harvested with RIPA lysis buffer (Sigma, St Louis, MO, USA) and quantified using a BCA protein assay kit (Invitrogen; Thermo Fisher Scientific, Inc.). Protein fractions were then separated via 10% SDS-PAGE and blotted onto PVDF membranes (EMD Millipore). Next, the membranes were blocked with 5% skim milk in PBST (0.2% Tween 20) for 2 h and incubated with primary antibodies against cleaved caspase 3 (cat. no. ab2302; 1:1000 dilution, Abcam, Cambridge, MA, USA), caspase 3 (cat. no. ab32351; 1:1000 dilution, Abcam, Cambridge, MA, USA), aggrecan (cat. no. ab194594; 1:1000 dilution, Abcam, Cambridge, MA, USA), collagen type II (cat. no. ab34712; 1:1000 dilution, Abcam, Cambridge, MA, USA), and GAPDH (cat. no. ab9485; 1:1000 dilution, Abcam, Cambridge, MA, USA). After washing, the membranes were incubated with the corresponding secondary antibody (cat. no. ab7090/ab7068; 1:2000 dilution, Abcam, Cambridge, MA, USA) for 1 h. Finally, the target bands were detected using ECL detection reagents (GE Healthcare Life Sciences, Beijing, China) according to the manufacturer’s instructions [31].

### Statistical analysis

SPSS software version 20.0 (SPSS Inc., Chicago, IL, USA) was used for statistical analyses. All results are expressed as the mean ± SD of values from three independent experiments. The differences between two groups were evaluated using Student’s t-test, and comparisons between multiple groups were analyzed using one-way analysis of variance (ANOVA) followed by Tukey’s post hoc test. Statistical significance was set at p < 0.05.

## Results

### Effect of ACVR1 on LPS-treated human NP cells

**Figure 1.**
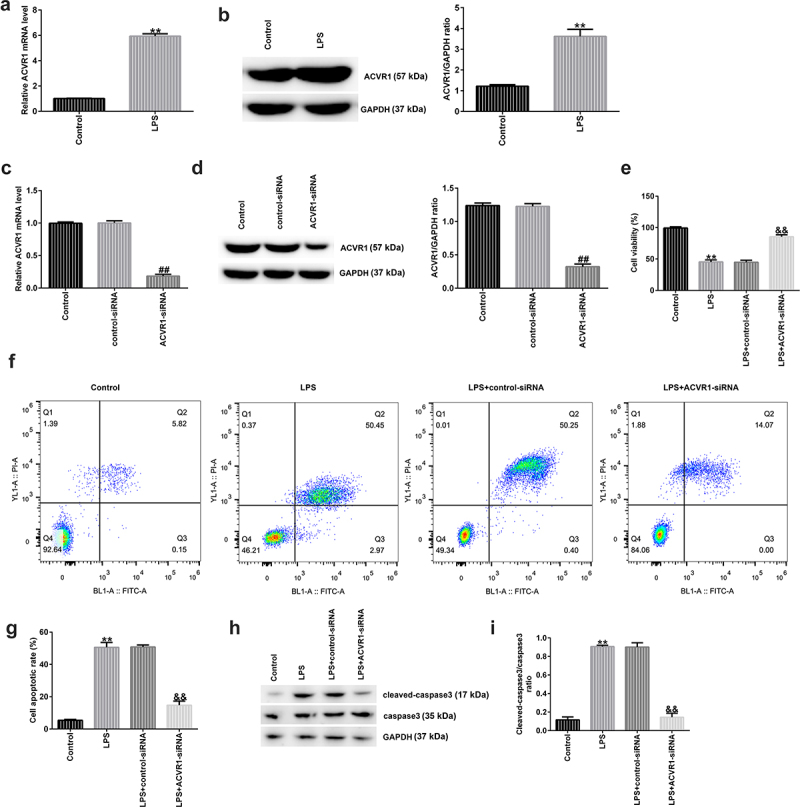
Silencing ACVR1 promotes cell growth and reduces apoptosis in LPS-exposed NP cells. (a and b) Detection of ACVR1 levels in the control and LPS groups through RT-qPCR and Western blotting. (c and d) RT-qPCR and Western blot analysis of ACVR1 in control-siRNA or ACVR1-siRNA-transfected NP cells. (e) NP cell viability in different groups was detected using the MTT assay. (f) Flow cytometric analysis of apoptotic cells. (g) Percentage of apoptotic cells in control-siRNA or ACVR1-siRNA-treated NP cells. (h) Expression of apoptosis-related proteins in NP cells, as detected using Western blot assay. (i) Cleaved-caspase 3/caspase 3 ratio. **p < 0.01 vs. control; ##p < 0.01 vs. control-siRNA; &&p < 0.01 vs. LPS+control-siRNA.

A recent study revealed that ACVR1 is markedly overexpressed in patients with IDD [10], but the specific mechanism needs to be further explored. To confirm whether ACVR1 participates in the progression of IDD, ACVR1 expression in NP cells was assessed using RT-qPCR and Western blotting. It was observed that ACVR1 was upregulated in LPS-induced degenerative NP cells (Figure 1(a,b)). These findings suggest that ACVR1 may be involved in the progression of IDD. Several functional assays were performed to explore the effects of ACVR1 on NP cells. NP cells were treated with control-siRNA or ACVR1-siRNA for 24 h, followed by LPS for another 24 h. It was observed that ACVR1 was downregulated in ACVR1-siRNA-transfected NP cells compared to that in the control-siRNA group (Figure 1(c,d)). In addition, the results shown in Figure 1(e) demonstrate that cell viability in the LPS group was considerably suppressed. Furthermore, Figure 1(f,g) show that LPS induced apoptosis in NP cells compared to that in the control cells. However, the opposite effects were observed in the ACVR1-siRNA group.

The expression of apoptosis-related proteins was determined via Western blotting. There was an obvious upregulation of cleaved caspase 3 expression and the cleaved-caspase 3/caspase 3 ratio in the LPS group, and these effects were reversed by ACVR1-siRNA (Figure 1(h,i)).

### Effects of ACVR1 Downregulation on Inflammatory Cytokine Production and ECM Degradation in the LPS-exposed NP Cells

**Figure 2.**
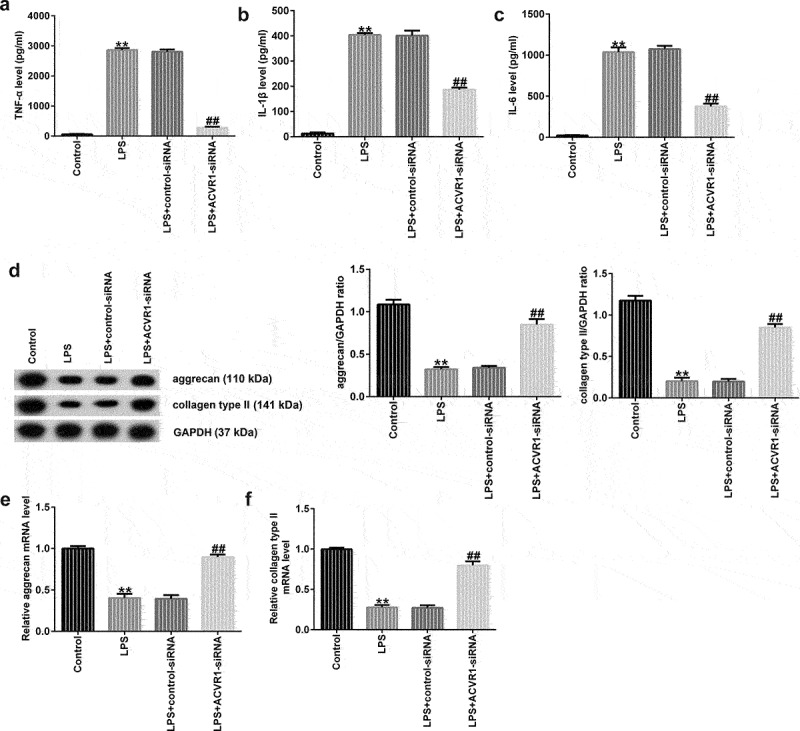
ACVR1-siRNA decreases inflammatory cytokine production and accelerates ECM degradation in NP cells after LPS treatment. NP cells were induced with control-siRNA or ACVR1-siRNA for 24 h, followed by 10 ng/mL LPS treatment. ELISA was used to determine the secretion levels of (a) TNF-α, (b) IL-1β, and (c) IL-6 in the different groups. (d) Western blot assay of aggrecan and collagen type II in the different groups. (e and f) Levels of ECM-associated genes (aggrecan and collagen type II) in the different groups were visualized via RT-qPCR. **p < 0.01 vs. control; ##p < 0.01 vs. LPS+control-siRNA.

Inflammation plays a key role in IDD development. Here, ACVR1 was downregulated by ACVR1-siRNA in NP cells, followed by LPS treatment for 24 h. ELISA results showed that LPS promoted the secretion of associated inflammatory factors (TNF-α, IL-1β, and IL-6), while ACVR1 suppression remarkably inhibited the LPS-challenged enhanced inflammatory factor expression in NP cells (Figure 2(a-c)). ECM degradation is also considered to be a vital marker for assessing NP cell function. Western blotting and RT-qPCR analyses revealed a reduction in ECM-related protein and mRNA expression in LPS-treated NP cells relative to that in the control group. Moreover, we observed opposite results in the LPS+ACVR1-siRNA group (Figure 2(d–f)). Our findings suggest that ACVR1 regulates inflammatory cytokine levels and ECM degradation in NP cells.

### ACVR1 is negatively regulated by miR-137 in human NP cells

**Figure 3.**
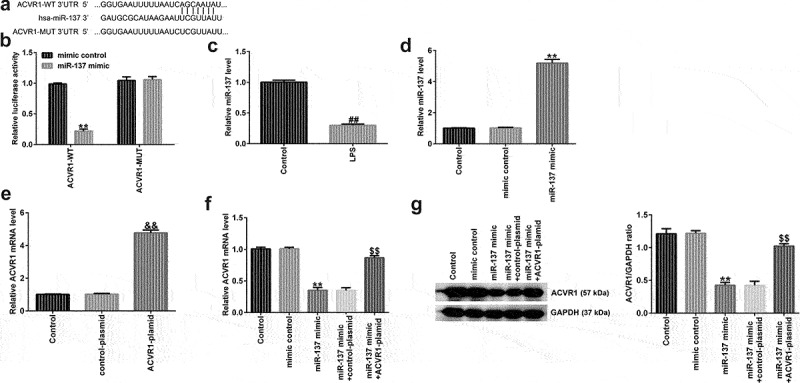
ACVR1 directly targets miR-137 and negatively regulates miR-137 expression in human NP cells. (a) Putative miR-137 binding sites in the 3ʹ-UTR of ACVR1, assessed using TargetScan. (b) Luciferase reporter assay results indicated an inter-association between ACVR1 and miR-137. (c) Measurement of miR-137 levels in the control and LPS groups via RT-qPCR. (d) RT-qPCR analysis of miR-137 mRNA levels in the mimic-control and miR-137 mimic groups. (e) RT-qPCR analysis of ACVR1 expression in NP cells after control-plasmid and ACVR1-plasmid treatments. (f and g) mRNA and protein levels of ACVR1 in the different groups. **p < 0.01 vs. mimic control; ##p < 0.01 vs. control; &&p < 0.01 vs. control-plasmid; $$p < 0.01 vs. miR-137 mimic+control-plasmid.

To further demonstrate the potential mechanism of ACVR1 in IDD, bioinformatic analysis was conducted using TargetScan version 7.2, and our study revealed that ACVR1 is a latent target gene of miR-137 (Figure 3(a)). miRNA-137 is reportedly implicated in various diseases [18–20]; a previous report suggested that miR-137 is downregulated in LPS-induced chondrocytes and articular cartilage samples, and that miR-137, via the AMPK/NF-κB signaling pathway, prevents TCF4 from reversing the progression of osteoarthritis [21]. Nevertheless, the biological function and latent mechanism of miR-137 in IDD remain unclear. Here, we hypothesized that miR-137 participates in IDD occurrence and progression by regulating the expression of ACVR1. To test this hypothesis, we used dual-luciferase reporter analysis to confirm whether miR-137 directly targets the 3ʹ-UTR of ACVR1. As shown in Figure 3(b), ACVR1 3ʹ-UTR WT luciferase activity was effectively reduced, whereas there was no obvious change in ACVR1-MUT luciferase activity, which suggested that ACVR1 directly targets miR-137. In addition, miR-137 levels in IDD were detected using RT-qPCR. We found that miR-137 was downregulated in LPS-treated NP cells (Figure 3(c)).

To explain the relationship between miR-137 and ACVR1 in NP cells, NP cells were treated with the mimic-control, miR-137 mimic, control-plasmid, or ACVR1-plasmid for 24 h. The results from RT-qPCR showed that miR-137 was upregulated in NP cells after miR-137 mimic transfection, compared to that in the mimic control group (Figure 3(d)). Furthermore, as indicated in Figure 3(e), after ACVR1-plasmid transfection, the levels of ACVR1 were increased in the control plasmid group. Furthermore, it was found that the upregulation of miR-137 led to a decrease in ACVR1 levels, while this effect was abolished after ACVR1-plasmid transfection (Figure 3(f,g)). These observations indicated that miR-137 negatively regulated ACVR1 expression in IDD.

### ACVR1-plasmid abolishes the effects of mir-137 mimic on cell viability and apoptosis after LPS stimulation

**Figure 4.**
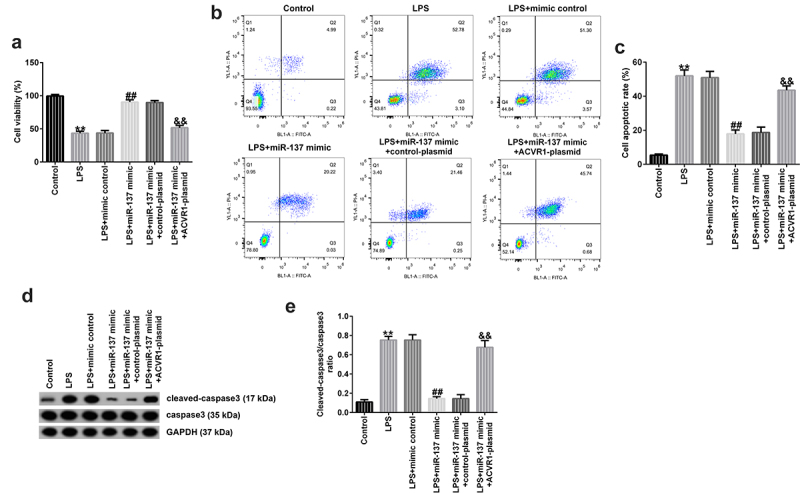
ACVR1-plasmid eliminates the effects of miR-137 mimic on NP cell growth and apoptosis. NP cells were transfected with the mimic-control, miR-137 mimic, control-plasmid, or ACVR1-plasmid, followed by 10 ng/mL of LPS. (a and b) Cell viability and apoptosis in the different groups were confirmed via the MTT assay and flow cytometric analysis, respectively. (c) Quantification of apoptotic NP cells. (d) Expression of apoptosis-associated proteins was confirmed in different groups through Western blot analysis. (e) Ratio of cleaved-caspase 3/caspase 3. **p < 0.01 vs. control; ##p < 0.01 vs. LPS+mimic control; &&p < 0.01 vs. LPS+miR-137 mimic+control-plasmid.

We further explored the influence of ACVR1 and miR-137 on NP cell function and investigated the association between them. NP cells were treated with the mimic-control, miR-137 mimic, control-plasmid, or ACVR1-plasmid for 24 h, followed by treatment with 10 ng/mL LPS. As shown in Figure 4(a), compared with that in the LPS group, NP cell viability was markedly enhanced in the miR-137 mimic group, and this effect was eliminated in the LPS+miR-137 mimic+ACVR1-plasmid group. In addition, the apoptotic rate was suppressed after miR-137 mimic transfection, and the opposite result was observed in the LPS+miR-137 mimic+ACVR1-plasmid group (Figure 4(b,c)). Moreover, Western blot analysis revealed that the miR-137 mimic noticeably reduced cleaved-caspase 3 levels and the cleaved-caspase 3/caspase 3 ratio. However, the opposite effects were observed in miR-137 mimic+ACVR1-plasmid co-transfected cells. Our data revealed that the ACVR1-plasmid abolished the effects of miR-137 mimic on the viability and apoptosis of LPS-treated NP cells.

### Upregulation of ACVR1 reversed the influence of mir-137 mimic on inflammatory factor secretion in LPS-Treated NP cells

**Figure 5.**
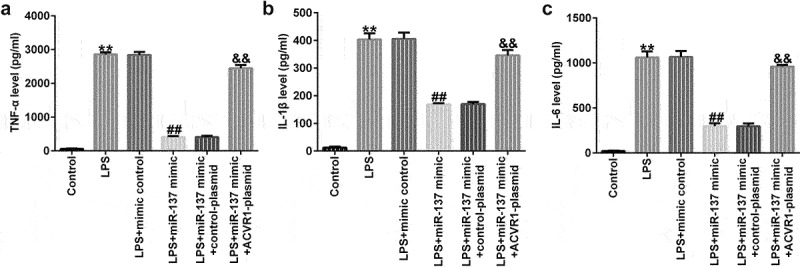
ACVR1-plasmid abolishes the effects of the miR-137 mimic on inflammatory factor secretion in NP cells. NP cells were exposed to the mimic-control, miR-137 mimic, control-plasmid, or ACVR1-plasmid, followed by 10 ng/mL of LPS. The secretion of inflammatory cytokines, including (a) TNF-α, (b) IL-1β and (c) IL-6, was visualized using ELISA. **p < 0.01 vs. control; ##p < 0.01 vs. LPS+mimic control; &&p < 0.01 vs. LPS+miR-137 mimic+control-plasmid.

We evaluated the effects of miR-137 and ACVR1 on the release of inflammatory factors in LPS-induced NP cells. ELISA results revealed that LPS significantly enhanced the secretion of inflammatory factors (TNF-α, IL-1β, and IL-6) in NP cells compared to that in the control. In addition, the miR-137 mimic suppressed TNF-α, IL-1β, and IL-6 levels; however, these results were reversed by the ACVR1-plasmid (Figure 5(a–c)), revealing that the inflammatory response is related to IDD.

### ACVR1-plasmid reverses the influence of mir-137 mimic on ECM metabolism in LPS-induced NP cells

**Figure 6.**
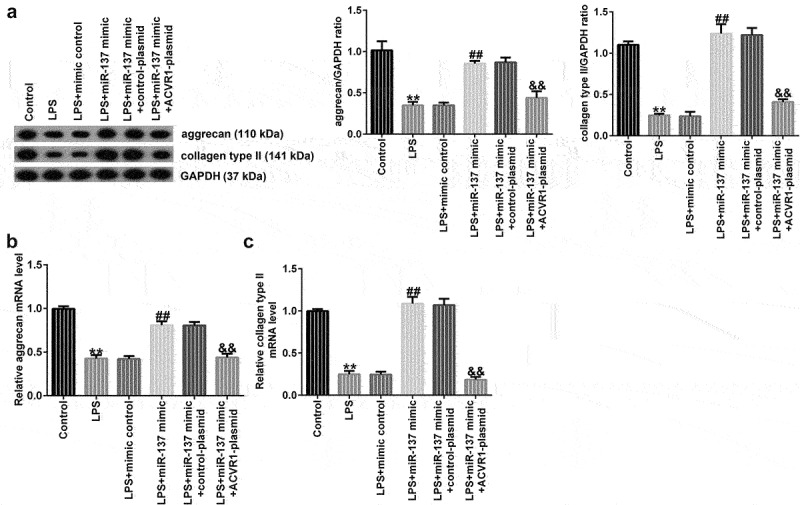
ACVR1-plasmid reverses the effects of the miR-137 mimic on ECM degradation in NP cells. NP cells were exposed to the mimic-control, miR-137 mimic, control-plasmid, or ACVR1-plasmid for 24 h, followed by 10 ng/mL LPS treatment. Aggrecan and collagen type II (a) protein and (b and c) mRNA expression levels in NP cells in the different groups were evaluated through (A) Western blotting and (B and C) RT-qPCR, respectively. **p < 0.01 vs. control; ##p < 0.01 vs. LPS+mimic control; &&p < 0.01 vs. LPS+miR-137 mimic+control-plasmid.

Next, we investigated whether miR-137 affects ECM degradation by targeting ACVR1. NP cells were induced with the mimic-control, miR-137 mimic, control-plasmid, or ACVR1-plasmid for 24 h, followed by 10 ng/mL LPS. According to the results of Western blot analysis, miR-137 mimic remarkably increased the aggrecan and collagen type II protein levels, while these levels were reduced by the ACVR1-plasmid. Similar results were observed with RT-qPCR, which showed that aggrecan and collagen type II mRNA expression was significantly enhanced in the LPS+miR-137 mimic group. However, this result was reversed by the ACVR1-plasmid (Figure 6(a–c)). Together, these findings suggested that miR-137 participates in ECM degradation by regulating ACVR1.

## Discussion

IDD is a common musculoskeletal disorder characterized by long-term back, neck, and lumbar dysfunction, which influences the quality of life of approximately 80% of patients with IDD [32,33]. Over the past few years, several studies have revealed that various risks, including age, may lead to IDD [34]. To date, clinical therapies for IDD are limited to relieving symptoms, while the detailed etiology of IDD needs to be further explored. Thus, the present study was designed to identify novel targets and explain the complicated mechanisms of IDD treatment. It is well known that LPS is widely applied to generate in vitro IDD models [22–24]. Therefore, in our study, 10 ng/mL LPS was used to induce NP cells to establish IDD models in vitro.

ACVR1 (also known as bone morphogenic protein receptor kinase activin A receptor), a vital receptor of BMP7, participates in the regulation of inflammation and cellular functions [35]. Previous reports have revealed that ACVR1 levels are higher in IDD patients than in healthy volunteers [10]; however, the molecular mechanism and role of ACVR1 in IDD remain unclear. We observed that ACVR1 was upregulated in LPS-induced NP cells compared to that in normal NP cells, confirming that ACVR1 might be associated with IDD progression. IDD is related to NP cell functions, such as growth, migration, and apoptosis [6]. Next, we investigated the role of ACVR1 in NP cell functions. To this end, NP cells were transfected with control siRNA or ACVR1-siRNA for 24 h and exposed to 10 ng/mL LPS for an additional 24 h. We found that ACVR1-siRNA inhibited ACVR1 expression in NP cells. Cell proliferation and apoptosis were determined using the MTT assay and flow cytometry. A reduction in cell growth and promotion of apoptosis were observed in LPS-stimulated cells. Furthermore, Western blot analysis revealed that the levels of cleaved caspase 3 were increased, while those of pro-caspase3 were reduced in LPS-exposed NP cells. However, the opposite effects were observed in the ACVR1-siRNA+LPS group compared with those in the control siRNA +LPS group.

Inflammation is a major pathogenic factor in IDD. Previous studies have confirmed that multiple inflammatory cytokines, including IL-6, TNF-α, and IL-1β, are overexpressed in disc tissue [36]. The present study further assessed the secretion of inflammatory factors by NP cells using ELISA. Our data indicated that LPS enhanced inflammatory cytokine secretion, which was markedly reduced after ACVR1-siRNA treatment. It is well known that reduction in NP cell number and ECM degradation are two primary features of IDD, which could lead to alterations in the fundamental architecture and maturation of the disc. ECM degradation products may result in increased IL-1β and TNF-α release [37]. Therefore, the effects of ACVR1-siRNA on ECM degradation were detected in LPS-induced NP cells. The results revealed that ACVR1-siRNA inhibited LPS-induced ECM degradation in LPS-induced NP cells. Our findings demonstrated that ACVR1 is involved in IDD progression through inflammatory responses and ECM degradation.

Numerous miRNAs have been shown to be associated with the progression of human diseases, such as IDD, via binding to the 3ʹ-UTR. Previous studies have shown that multiple miRNAs, including miR-138-5p [38], miR-24-3p [39] and miR-640 [40], are abnormally expressed in IDD. For instance, miR-24-3p induces human IDD by regulating insulin-like growth factor-binding proteins and activating the ERK signaling pathway. However, the specific roles and underlying mechanisms of miRNAs have not yet been fully elucidated. Bioinformatic databases were analyzed to predict the association between ACVR1 and miRNAs, and it was suggested that ACVR1 directly targets miR-137.

miR-137, a newly identified miRNA, has been studied in various diseases. Moreover, miR-137 has been shown to be involved in the regulation of inflammation, ECM, and cell function [41–43]. Nevertheless, the precise mechanism of action of miR-137 in IDD remains unknown. In the present study, it was found that miR-137 was downregulated in NP cells after LPS treatment. These results suggested that miR-137 directly targets ACVR1 and negatively regulates ACVR1 expression in IDD. It can be speculated that miRNA-137 may be related to the progression of IDD and may play a vital role in regulating IDD pathogenesis. The association between miR-137 and ACVR1 in NP cells was assessed using functional analyses. We confirmed that the miR-137 mimic enhanced NP cell growth and reduced apoptosis following LPS stimulation. Moreover, we investigated whether miR-137 regulates the inflammatory response and ECM degradation in LPS-induced NP cells. Indeed, the results revealed that miR-137 mimic suppressed the inflammatory response and enhanced the levels of aggrecan and collagen type II in LPS-treated NP cells; nevertheless, all these results were inversed after ACVR1-plasmid transfection. Together, these data suggested that ACVR1 overexpression halted the effects of miR-137 mimic on LPS-treated NP cells. However, the function of miR-137 in IL-1β-treated NPCs or damaged NPCs in IDD patients has not been explored, which is a limitation of this study. Another limitation of this study was that only aggrecan and collagen type II levels were measured for ECM detection, and the levels of MMPs were not determined.

However, it is necessary to explore more regulators associated with the progression of IDD and investigate new therapeutic methods against IDD. To clarify the functions of ACVR1 and miR-137 in IDD, multiple in-depth experiments are required. For instance, the expression of ACVR1 and miR-137 in the NP tissue/serum of patients with IDD needs to be clarified. Moreover, the mechanisms by which ACVR1 regulates caspase 3 cleavage and affects NP cell viability and apoptosis need to be clarified. In addition, the roles of ACVR1 and miR-137 in IDD should be investigated *in vivo*. These issues will be addressed in our future research.

## Conclusion

This is the first report to reveal an important association between miR-137 and ACVR1 in NP cells. Our findings demonstrated the protective effects of miR-137 on LPS-treated NP cells, as evidenced by inhibition of the inflammatory response and ECM degradation via the regulation of ACVR1. Our findings suggest that miRNA-137 is involved in the progression of IDD by targeting ACVR1.

## Supplementary Material

Supplemental Material

## Data Availability

The datasets used and/or analyzed during the current study are available from the corresponding author on reasonable request.

## Research highlights

ACVR1 downregulation reduces cell apoptosis, decreases inflammatory cytokine production and ECM degradation in the LPS-exposed NP cells;

ACVR1 is negatively regulated by miR-137 in human NP cells;

ACVR1-Plasmid abolishes the effects of miR-137 mimic on LPS-exposed NP cells.
