# Supplementary figures and images for: MicroRNA-137 inhibits the inflammatory response and extracellular matrix degradation in lipopolysaccharide-stimulated human nucleus pulposus cells by targeting activin a receptor type I
Source: Bioengineered. 2022 Mar 2;13(3):6396–408. doi: 10.1080/21655979.2022.2042987 (PMC8973860; doi:10.1080/21655979.2022.2042987)

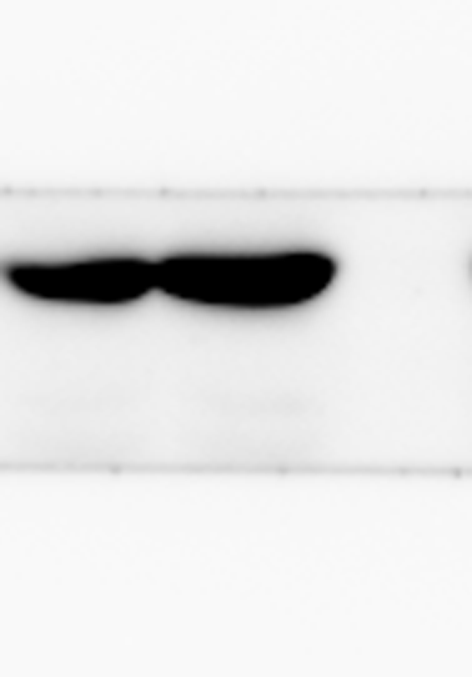

Supplement: Supplemental Material [file KBIE_A_2042987_SM4812.zip › supplementary data/original blots/original blots Figure1/ACVR1(B).tif]

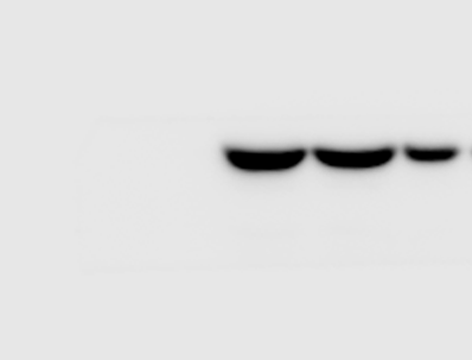

Supplement: Supplemental Material [file KBIE_A_2042987_SM4812.zip › supplementary data/original blots/original blots Figure1/ACVR1(D).tif]

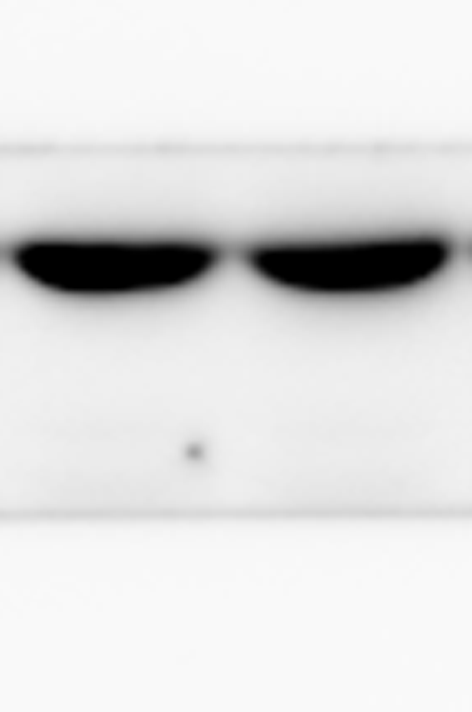

Supplement: Supplemental Material [file KBIE_A_2042987_SM4812.zip › supplementary data/original blots/original blots Figure1/GAPDH(B).tif]

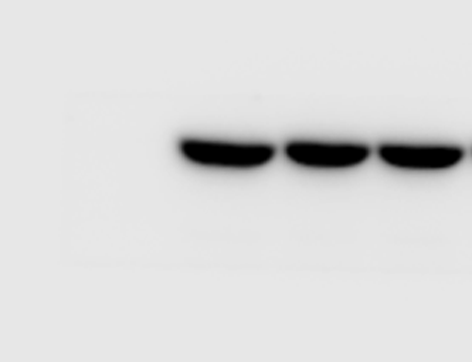

Supplement: Supplemental Material [file KBIE_A_2042987_SM4812.zip › supplementary data/original blots/original blots Figure1/GAPDH(D).tif]

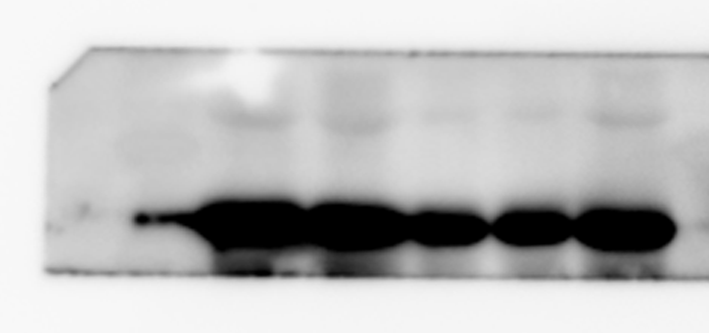

Supplement: Supplemental Material [file KBIE_A_2042987_SM4812.zip › supplementary data/original blots/original blots Figure3/ACVR1.tif]

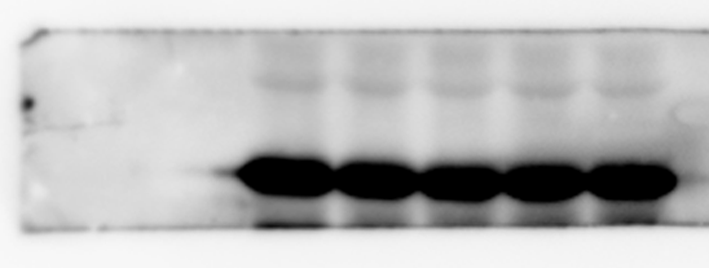

Supplement: Supplemental Material [file KBIE_A_2042987_SM4812.zip › supplementary data/original blots/original blots Figure3/GAPDH.tif]

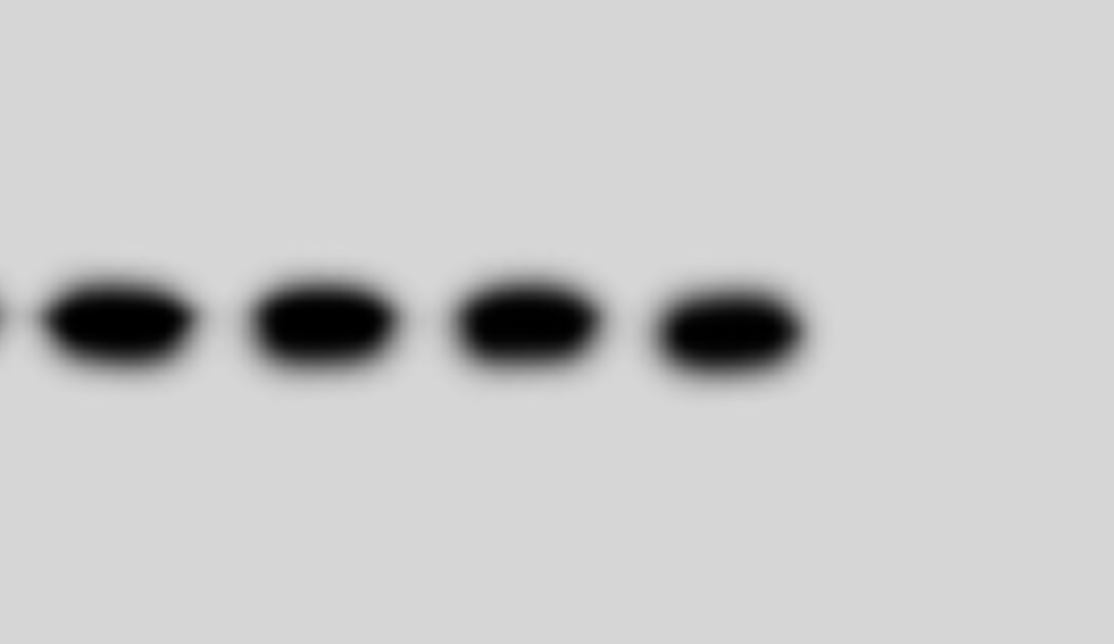

Supplement: Supplemental Material [file KBIE_A_2042987_SM4812.zip › supplementary data/original blots/original blots-Figure 2/GAPDH.tif]

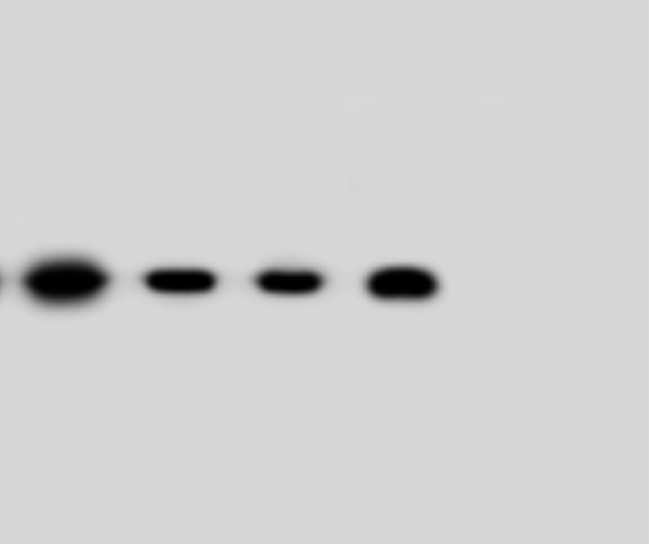

Supplement: Supplemental Material [file KBIE_A_2042987_SM4812.zip › supplementary data/original blots/original blots-Figure 2/aggrecan.tif]

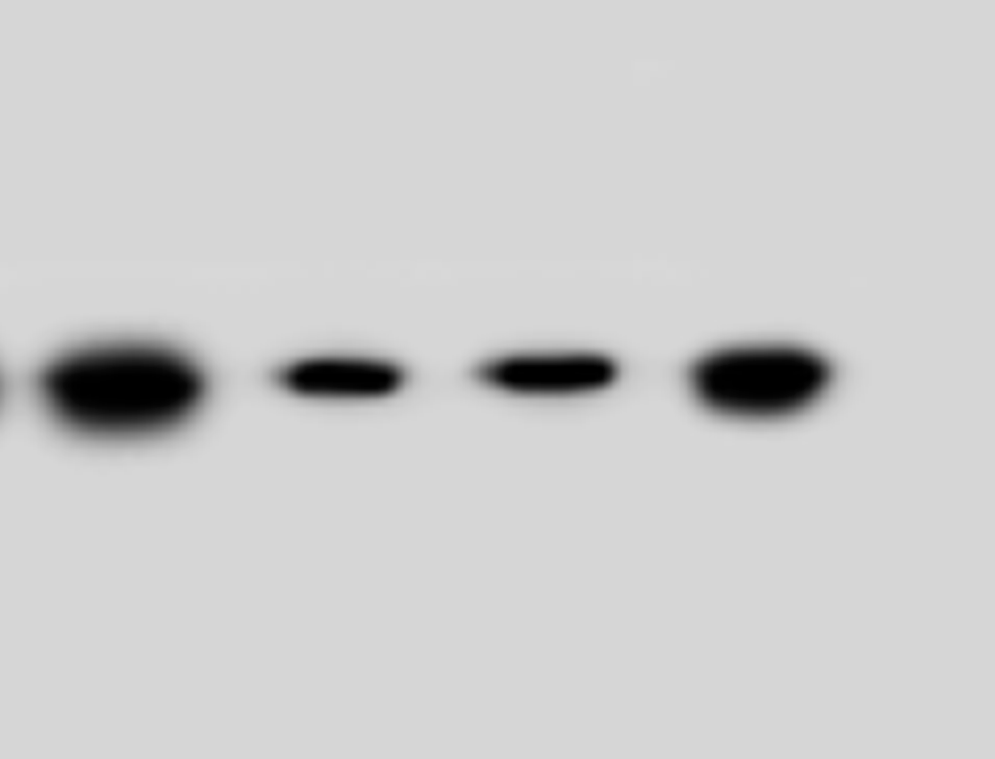

Supplement: Supplemental Material [file KBIE_A_2042987_SM4812.zip › supplementary data/original blots/original blots-Figure 2/collagen type II.tif]

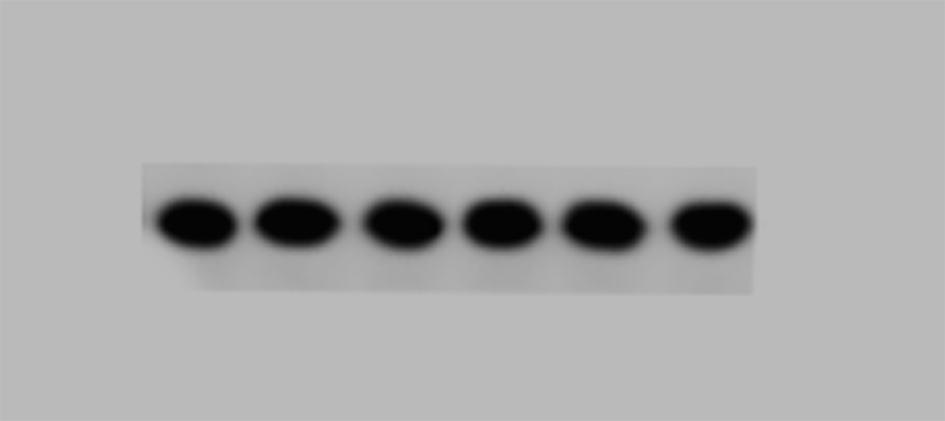

Supplement: Supplemental Material [file KBIE_A_2042987_SM4812.zip › supplementary data/original blots/original blots-Figure4/Caspase3.tif]

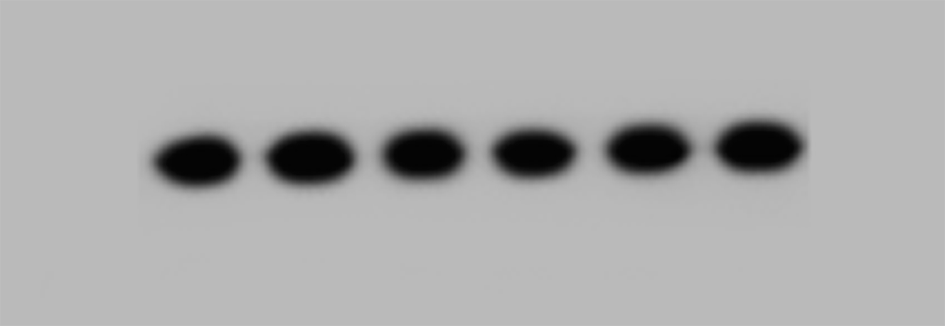

Supplement: Supplemental Material [file KBIE_A_2042987_SM4812.zip › supplementary data/original blots/original blots-Figure4/GAPDH.tif]

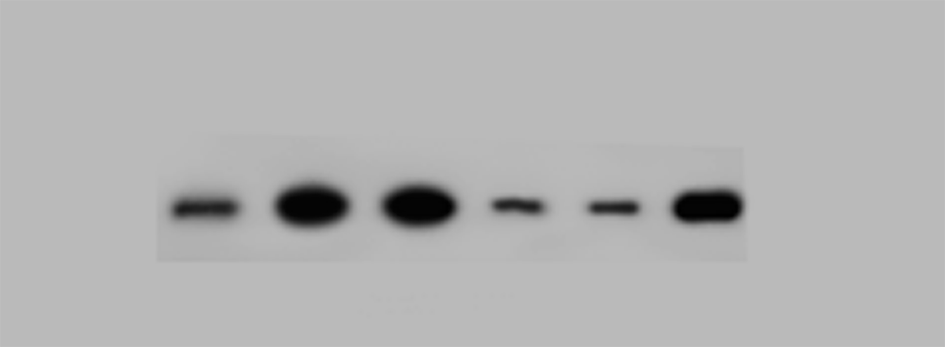

Supplement: Supplemental Material [file KBIE_A_2042987_SM4812.zip › supplementary data/original blots/original blots-Figure4/cleaved-Caspase3.tif]

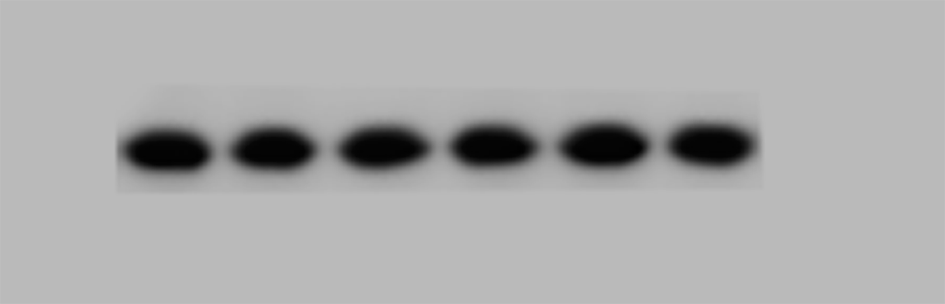

Supplement: Supplemental Material [file KBIE_A_2042987_SM4812.zip › supplementary data/original blots/original blots-Figure6/GAPDH.tif]

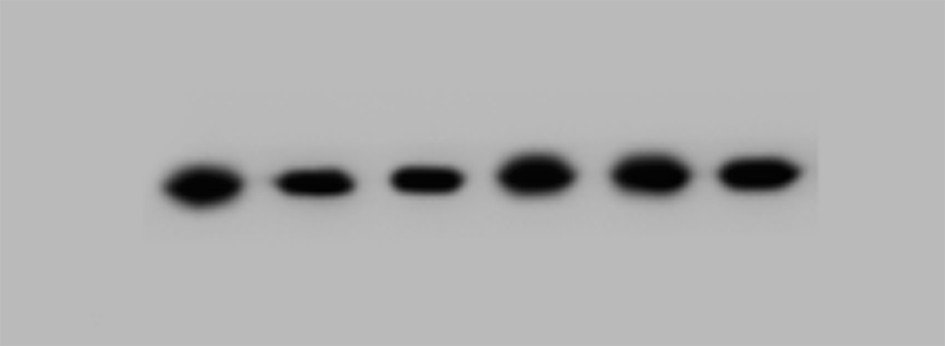

Supplement: Supplemental Material [file KBIE_A_2042987_SM4812.zip › supplementary data/original blots/original blots-Figure6/aggrecan.tif]

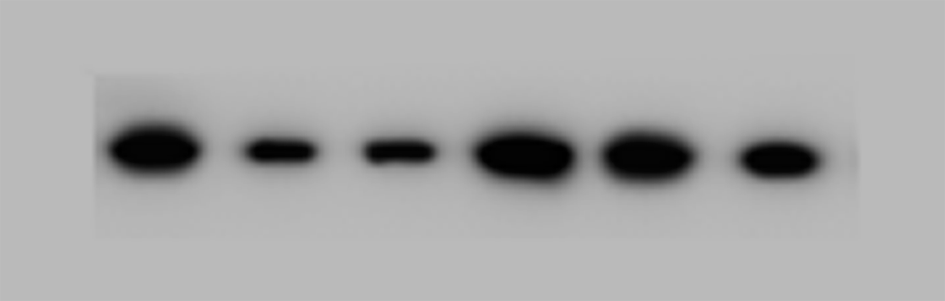

Supplement: Supplemental Material [file KBIE_A_2042987_SM4812.zip › supplementary data/original blots/original blots-Figure6/collagen type11.tif]
