# Supplementary material for: MicroRNA-137 inhibits the inflammatory response and extracellular matrix degradation in lipopolysaccharide-stimulated human nucleus pulposus cells by targeting activin a receptor type I
Source: Bioengineered. 2022 Mar 2;13(3):6396–408. doi: 10.1080/21655979.2022.2042987 (PMC8973860; doi:10.1080/21655979.2022.2042987)
Supplement: Supplemental Material [file KBIE_A_2042987_SM4812.zip › supplementary data/original blots/Notes.docx]

Dear editor,

  Thank you very much for considering our manuscript. The original blots of proteins have been provided. However, it should be noted that during the Western blot assay, we first cut out the corresponding membrane according to the molecular weight of the target protein and then incubate the primary antibody. Therefore our original imprint is not a full film. However, we guarantee that all our experiments are carried out scientifically and strictly in accordance with the experimental procedures. Hope to get your understanding.

Best regards
